# Laser Remelting Process Simulation and Optimization for Additive Manufacturing of Nickel-Based Super Alloys

**DOI:** 10.3390/ma15010177

**Published:** 2021-12-27

**Authors:** Fabian Soffel, Yunong Lin, Dominik Keller, Sergei Egorov, Konrad Wegener

**Affiliations:** 1 Inspire AG, Technoparkstrasse 1, 8005 Zürich, Switzerland; 2 Institute of Machine Tools and Manufacturing, ETH Zürich, Leonhardstrasse 21, 8092 Zürich, Switzerland; yunlin@student.ethz.ch (Y.L.); keller@iwf.mavt.ethz.ch (D.K.); egorov@iwf.mavt.ethz.ch (S.E.); wegener@iwf.mavt.ethz.ch (K.W.)

**Keywords:** additive manufacturing, laser remelting, direct metal deposition, process modeling, repair welding, Inconel, process chain

## Abstract

Nickel-based super alloys are popular for applications in the energy and aerospace industries due to their excellent corrosion and high-temperature resistance. Direct metal deposition (DMD) of nickel alloys has reached technology readiness for several applications, especially for the repair of turbomachinery components. However, issues related to part quality and defect formation during the DMD process still persist. Laser remelting can effectively prevent and repair defects during metal additive manufacturing (AM); however, very few studies have focused on numerical modeling and experimental process parameter optimization in this context. Therefore, the aim of this study is to investigate the effect of determining the remelting process parameters via numerical simulation and experimental analyses in order to optimize an industrial process chain for part repair by DMD. A heat conduction model analyzed 360 different process conditions, and the predicted melt geometry was compared with observations from a fluid flow model and experimental single tracks for selected reference conditions. Subsequently, the remelting process was applied to a demonstrator repair case. The results show that the models can well predict the melt pool shape and that the optimized remelting process increases the bonding quality between base and DMD materials. Therefore, DMD part fabrication and repair processes can benefit from the remelting step developed here.

## 1. Introduction

Metal additive manufacturing (AM) of nickel-based super alloys has been studied widely in recent years, primarily for potential applications in the energy and aerospace industries. While powder bed fusion (PBF) processes have various advantages for smaller and complex-shaped part geometries, the fabrication or repair of larger parts may require the increased machine size and accessibility of directed energy deposition (DED) processes. Direct metal deposition (DMD) is a process whereby a laser creates a melt pool into which metallic powder particles are continuously blown in order to create welding tracks. The repair of turbomachinery components is a typical application of DMD. One challenge in DMD is the formation of defects, especially in the transition zone between the substrate and the AM material. Laser remelting processes have been shown to significantly reduce both bonding defects and cracks [1]. Therefore, it is of high interest to include laser remelting steps in part fabrication and repair process chains of nickel-based super alloys which use DMD.

Laser remelting is a laser welding process with the purpose of local material melting and resolidification. In metal AM, laser remelting can be applied to improve part quality in terms of surface roughness, magnetic properties, and mechanical properties. Liu et al. [2] fabricated AlSi10Mg test structures by PBF; laser remelting reduced surface roughness by 25%, as previously unmelted or partially melted powder particles were fused with the deposit. Yang et al. [3] applied laser remelting to an iron–cobalt-based alloy deposited by PBF, and showed that saturation magnetization and microhardness increased due to grain refinement. Liu et al. [1] used laser remelting for the deposition of the nickel-based super alloy K417G by DMD. They found that remelting significantly reduced crack density within the DMD structure. For the repair of Inconel 718 and Waspaloy turbomachinery components, Liu et al. [4] proposed a combined process chain consisting of defect milling, sandblasting, laser remelting, and DMD. The laser remelting step was used to reduce liquation cracking in the heat-affected zone of the base material. The main parameters of the laser remelting processes are laser spot size, laser power, and scanning speed. It is expected that numerical simulation can support the determination of suitable laser remelting processing parameters for a specific application.

Numerical models for the simulation of laser welding and AM processes are characterized by the model domain, heat source definition, material properties, and the governing equations for heat transfer and fluid flow. The length scale of the model domain ranges from less than a millimeter for the analysis of melt pool dynamics [5] to several centimeters or even meters where the development of the temperature field on the part scale is of interest [6]. For melt pool modeling of a single welding track, the symmetry of the process permits consideration of only one half of the track within the domain, in order to reduce computational costs [7,8]. According to Wei et al. [9], heat sources can be defined as point or line heat sources for analytical models, and as surface or volumetric heat sources for numerical models. Wirth et al. [10] presented a surface heat source model that considered losses due to attenuation and reflection for a laser cladding process model applied to nickel- and cobalt-based alloys. DebRoy et al. [11] emphasized the importance of reliable material properties as a function of temperature. Because Inconel 718 is the most commonly-used nickel-based super alloy, several studies have focused on the determination of its material properties over a wide range of temperatures [12,13,14]. With these properties, the governing equations for heat transfer and fluid flow can be solved. The governing equations are based on the conservation of mass, momentum, and thermal energy and are mathematically described by the continuity, Navier–Stokes, and heat transfer equations, respectively. Process models typically involve various simplifications in order to reduce computational costs. According to DebRoy et al. [11], a common simplification is the neglection of convective heat transfer, which can be appropriate in conditions where no powder fusion occurs. As described by Wei et al. [9], the neglection of convective heat transfer can have a significant impact on the computed temperature distribution in the melt pool. Therefore, accurate prediction of time-dependent spatial temperature fields requires the consideration of fluid flow. However, for assessment of the sensitivity of the melt pool geometries to process conditions, pure heat conduction models may be sufficient.

The aim of the present study is to develop a numerical laser remelting model and to use the modeling results for the optimization of a laser remelting step within an industrial process chain. A heat conduction model was developed to study the effect of processing parameters on the melt pool geometry at low computational costs. A second model that considers the fluid flow in the melt pool was adapted, and both modeling results were compared to the experimental records. While the fluid flow model showed a higher physical accuracy and high computing times, the heat conduction model could be solved very quickly and with slight deviations of between +1.5 and +19.1% compared to the experimental observations. An optimized process chain including groove milling, laser remelting, and DMD was evaluated for cast Inconel 718 substrates. The laser remelting step led to a more uniform bonding of the DMD deposit to the cast substrate material compared to a combined process without intermediate surface remelting. The paper is structured as follows: Section 2 focuses on the numerical models and input parameters; Section 3 describes the experimental procedures; Section 4 presents and discusses the simulation and experimental results; and finally, Section 5 summarizes the main conclusions of the present study.

## 2. Numerical Modeling Approach

Two numerical models were developed in order to analyze the laser remelting process. The main difference between the two remelting models was the fluid flow within the melt pool, which was neglected in the heat conduction model in order to increase computational efficiency. Both approaches were based on the laser cladding process model presented by Wirth [15] and realized in COMSOL Multiphysics. The two modified models shared the same model domain, heat source definition, and material properties, which are described in the following sub-chapters.

### 2.1. Model Domain

**Figure 1:**
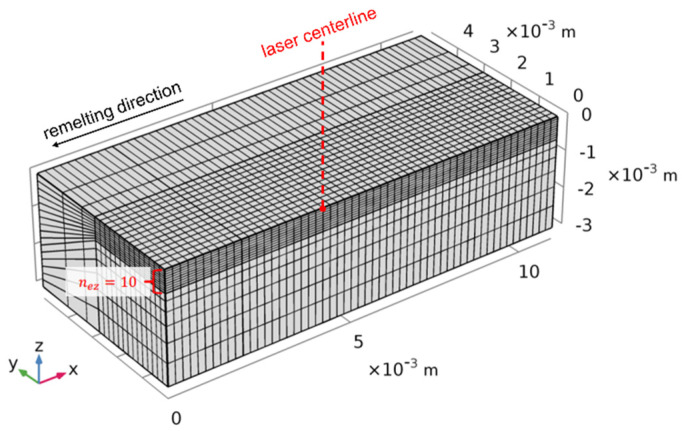
Mesh geometry and heat source location.

The rectangular model domain had a length of 9.00 mm in *x* direction, a width of 5.34 mm in *y* direction, and a height of 3.00 mm in *z* direction. The model was set up in the Eulerian coordinate system with a stationary heat source centered at *x* = 4.50 mm and *y* = 0 mm. As indicated in Figure 1, the substrate material flow was along the positive *x* axis, leading to a welding direction in the negative *x* direction. Due to the symmetry of the melt problem, only one half of the remelting process was modeled in order to save computational efforts. There was a fine-meshed zone in the area of the heat source where the number of elements in *z* direction *n_ez_* increases in order to enhance the spatial resolution of the computed temperature fields. Temperature profiles in the heat source center were used for a mesh convergence study.

### 2.2. Heat Source Definition

**Figure 2:**
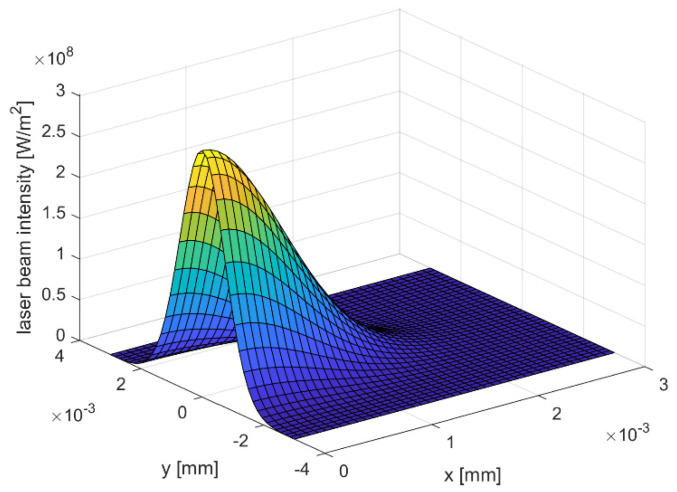
Laser beam intensity for spot size *s* = 3 mm.

The laser energy input was modeled as a surface heat source

(1) $$Q\left(x,y\right)=I\left(x,y\right)\cdot \alpha_{wp}$$

with the laser intensity *I(x*,*y*) and the work piece absorptivity *α_wp_*; the intensity distribution

(2) $$I\left(r\right)=\frac{2P}{\pi r_{L}{}^{2}}\exp\left(-2\frac{r^{2}}{r_{L}{}^{2}}\right),$$

considering the laser power *P* and the laser spot radius *r_L_*, is defined as a bivariate Gaussian distribution. Figure 2 illustrates the spatial laser beam intensity for the selected laser spot diameter *s* of 3 mm. Fixing the laser source at position *x* = 4.50 mm and *y* = 0 mm within the model domain, the radial distance *r* is expressed as follows:

(3) $$r\left(x,y\right)=\sqrt{{\left(x-4.5\right)}^{2}+y^{2}}$$

### 2.3. Heat Conduction Model

For this model a stationary solver was applied that solves the governing equations without time-dependent terms. In all zones of the domain the simplified heat transfer equation

(4) $$\frac{\partial \left(\rho c_{p}T\right)}{\partial t}+\nabla \cdot \left(-k\nabla T\right)=0$$

with density *ρ*, specific heat capacity *c_p_*, temperature *T*, and heat conductivity *k* is taken into account. The surface heat input is treated as a boundary condition of the heat conduction term at the upper surface of the model. Heat losses due to convective heat distribution and thermal radiation are neglected, as they were shown to have only a minor effect on the resulting melt pool geometries and their neglection increases the computational efficiency of the model [15]. The velocity field is set to be constant and uniform, such that all material is restricted to move only with the defined scanning speed *v* and the fluid flow within the melt pool is neglected.

### 2.4. Fluid Flow Model

The fluid flow model was built with a time-dependent solver and adaptive time stepping. Heat convection inside the domain is now considered in the heat transfer equation

(5) $$\frac{\partial \left(\rho c_{p}T\right)}{\partial t}+\rho c_{p}\underline{u}\cdot \nabla T+\nabla \cdot \left(-k\nabla T\right)=0$$

with the velocity vector $\underline{u}$. Fluid flow is described by the continuity equation

(6) $$\nabla \cdot \underline{u}=0$$

and the Navier–Stokes equation for incompressible flow

(7) $$\rho \left[\frac{\partial \underline{u}}{\partial t}+\underline{u}\cdot \left(\nabla ⊗\underline{u}\right)\right]=\nabla \left\{-p\underline{\underline{I}}+\mu \left[\nabla ⊗\underline{u}+{\left(\nabla ⊗\underline{u}\right)}^{Τ}\right]\right\}+\underline{F}$$

with pressure *p*, dynamic viscosity *μ*, identity tensor $\underline{\underline{I}}$ and volume force vector $\underline{F}$, by which the effects of gravity and buoyancy are considered. Surface tension and Marangoni stresses are taken into account by surface boundary conditions as described by Wirth [15].

### 2.5. Material Properties

The material properties for Inconel 718 used in the numerical study originate from various references. The constant parameters for the density *ρ* = 8190 kg/m^3^, solidus temperature *T_s_* = 1528 K, liquidus temperature *T_l_* = 1610 K, melting enthalpy *h_m_* = 227,000 J/kg, and the coefficient of thermal expansion *β* = 6.473 × 10^−5^ K^−1^ were taken from Pottlacher et al. [12]. The work piece absorptivity *α_wp_* was defined as a constant and set to 0.3 per Anderson et al. [16]. Table 1 summarizes the constant material properties.

**Figure 3:**
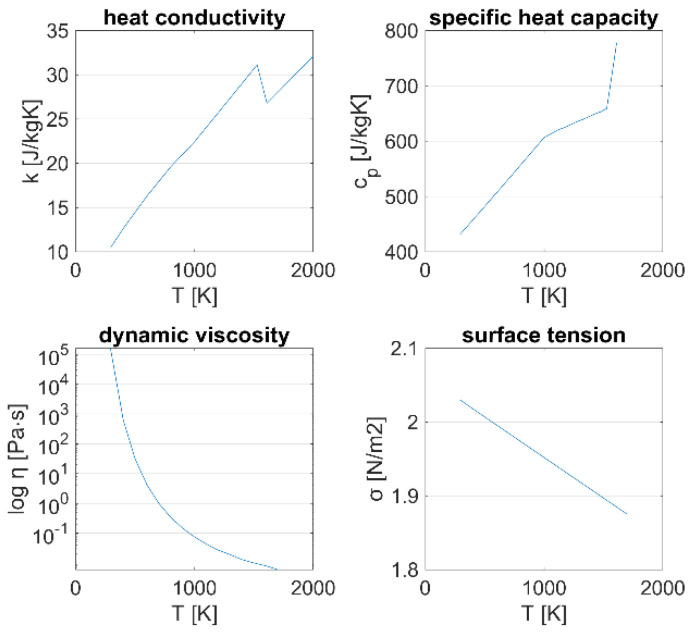
Temperature-dependent material properties of Inconel 718 from the COMSOL Multiphysics material library, Pottlacher et al. [12], Hosaeus et al. [13], and Overfelt et al. [14].

Figure 3 illustrates the temperature-dependent material properties. At temperatures that are higher than the specified values, the properties are treated as constants. The data for the heat conductivity *k* and isobaric specific heat capacity *c_p_* were taken from the material library of COMSOL Multiphysics for 293.15 K ≤ *T* ≤ 1000 K, and from Pottlacher et al. [12] and Hosaeus et al. [13] for *T* > 1000 K. The dynamic viscosity

(8) $$\eta \left(T\right)=0.000179e^{\left(\frac{50.2}{RT}\right)}\left(\mathrm{Pa}\cdot s\right)$$

is described by the Arrhenius equation, with the universal gas constant *R* = 0.008314 kJ/mol/K and the other constants as determined by Overfelt et al. [14] for Inconel 718. The surface tension

(9) $$\sigma \left(T\right)=1.842\frac{N}{m}-0.00011\frac{N}{mK}\left(T-1998.15\;K\right)$$

is defined as a linear function with a negative gradient based on the formula provided by Mills and Su [17].

## 3. Experimental Procedure

Within the experimental part of this study, single track tests were performed to validate the results of the numerical simulations. Subsequently, an optimized laser remelting process was developed for a groove repair application. The laser remelting and DMD experiments were carried out on a five-axis GF HPM 450 U milling machine with an Ambit S5 laser processing system. The system included an IPG fiber laser YLR-1000-MM-WC with a maximum laser power of 1000 W and wave length of 1070 nm. The rotary axes were fixed to their neutral position such that the developed processes could directly be transferred to any three-axis machine. The working distance of the laser head was set at 9.0 mm for DMD, as this is the distance with the highest powder efficiency and results in an approximate melt pool width of 2.2 mm. For the laser remelting step, the powder flow was deactivated and the working distance was increased to 15.0 mm in order to obtain an approximate melt pool width of 3.0 mm, which increases the efficiency of the process. Investment cast Inconel 718 cylinders in solution-annealed condition with a diameter of 26 mm and a height of 50 mm were utilized as substrates. The spherical powder material for the DMD experiments was Inconel 718 from LPW Technology Ltd., with a particle size distribution from 44 to 105 μm at a flow rate of 4.1 g/min. Argon was selected as the shielding, nozzle protection and powder carrier gas at flow rates of 8 L/min, 4 L/min, and 6 L/min, respectively.

**Figure 4:**
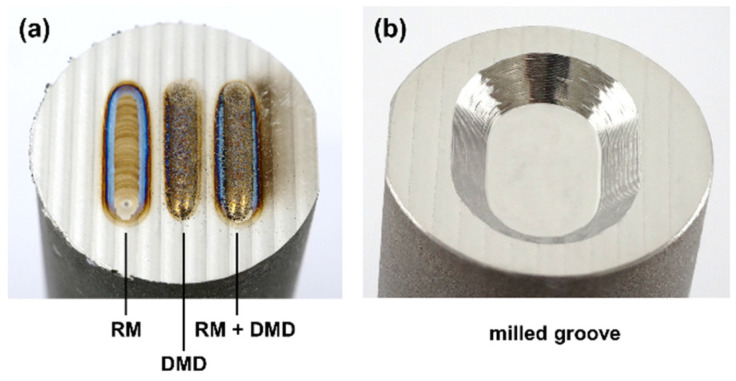
(**a**) Single tracks deposited in face-milled substrates and (**b**) groove geometry after machining.

**Figure 5:**
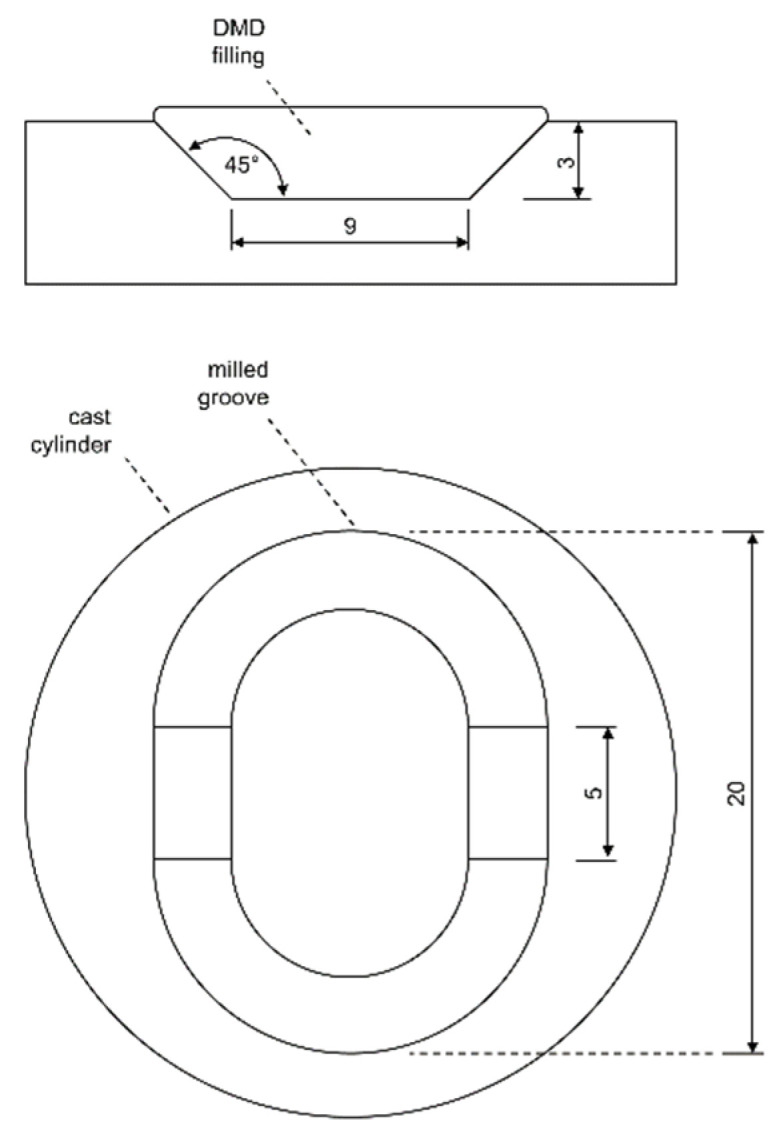
Dimensions of the groove geometry.

For the validation of the numerical models, 10 mm single remelting tracks were applied on face-milled substrates. A cooling time of more than 10 min was applied between each track in order to ensure that the substrate material was cooled to room temperature. For validation, a constant laser power of 1000 W and six levels of scanning speed from 150 to 400 mm/min in steps of 50 mm/min were used. Metallographic cross sections were prepared and evaluated in etched condition by optical microscopy and image analysis. To assess the effect of laser remelting on the subsequent DMD process, one DMD welding track with and without prior remelting was analyzed for one specimen, as illustrated in Figure 4a. To optimize the laser remelting process for repair applications a groove was milled into the cast substrates, as shown in Figure 4b; the groove geometry is illustrated in Figure 5. The milling tool for this process step was an end mill with a 6 mm diameter and a corner radius of 0.1 mm.

**Figure 6:**
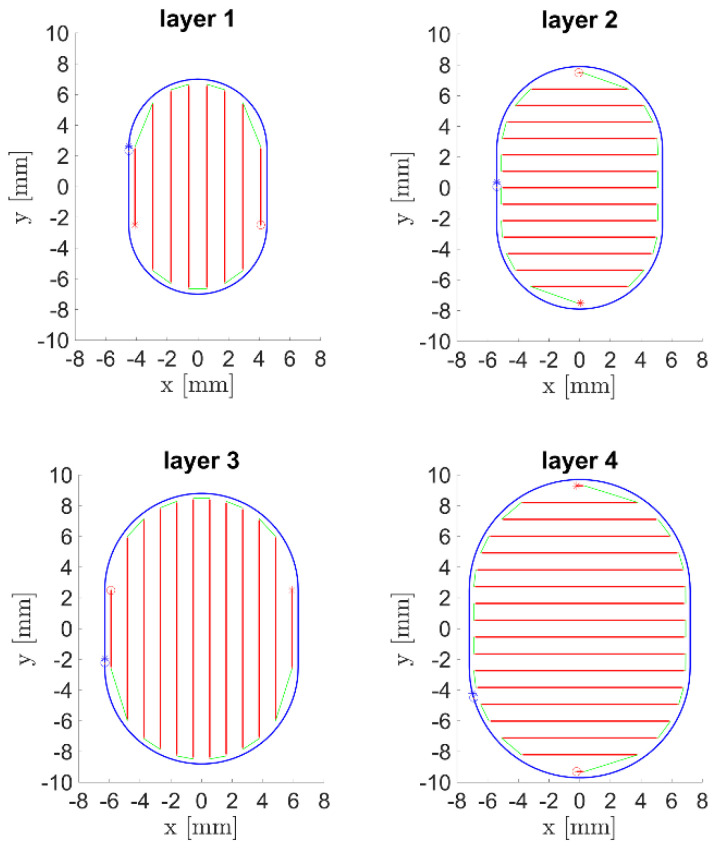
Laser tool path for the DMD groove fillings, with raster tracks in red, linking moves in green, and outer contour tracks in blue.

To calculate the laser tool paths, self-developed research CAM software by Eisenbarth [18] was used. For the DMD filling of the groove, four deposition layers with a height of 0.9 mm each were calculated with a contour/raster scanning strategy, as visualized in Figure 6. The contour to raster path distance was 0.4 mm, and the hatching distance between the raster paths was set to 1.1 mm. For the laser remelting process, the same tool path was applied for the first layer, and contour paths only for the following layers.

## 4. Results and Discussion

### 4.1. Simulation Results

The heat conduction model could be solved with a computation time of less than 30 s, and was therefore selected for analyzing the effects of the processing parameters on the resulting melt pool geometry. In total, 360 process conditions were solved by the heat conduction model within the scope of a full-factorial parameter study (Table 2). The fluid flow model was used to compute the velocity fields within the melt pool and its final dimensions for one reference condition, for which the computation time was over 16 h. The simulative results of both models were then compared to the experimental conditions.

**Figure 7:**
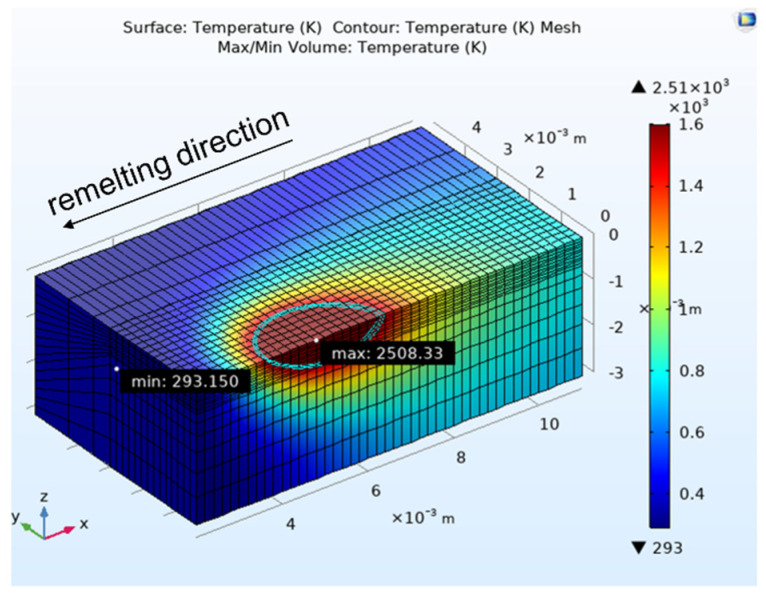
Stationary temperature field calculated by the heat conduction model with *P* = 1000 W, *v* = 400 mm/min, *T* = 20 °C.

Figure 7 shows the simulated stationary temperature field obtained by the heat conduction model for one reference condition. The isothermal curves visualize the boundaries of the predicted melt pool. The melt pool front experiences a larger temperature gradient than the backside, which leads to an elliptical melt pool shape. This finding is in agreement with the results of Turichin et al. [19], who modeled a laser welding process for DMD with and without powder addition for three materials (Inconel 718, stainless steel 316 L, and a titanium alloy) and found elongated melt pool geometries for all conditions. Hence, the results confirm that laser welding at moderate to high scanning speeds typically leads to elliptical melt pool shapes.

**Figure 8:**
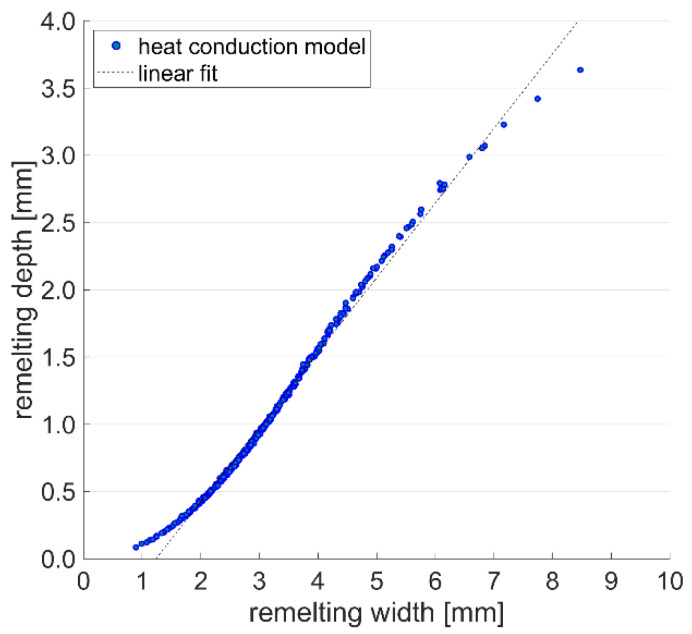
Correlation of remelting width and depth for the simulation results of the heat conduction model with the fit, according to Equation (10).

The geometrical correlation between remelting depth and width for all simulation results of the heat conduction model is illustrated in Figure 8. The ratio of remelting width to depth slowly decreases for larger melt pool geometries until a width of approximately 5 mm. For a wide range of modeling conditions, the linear approximation

(10) d=0.555w−0.6846

with a determination coefficient *R*^2^ of 0.992 leads to an accurate prediction of the remelting depth *d* from the modeled remelting width *w*. Thus, the effect of the processing parameters can be analyzed for one geometrical feature of the melt pool only, as the other feature can be deduced by linear approximation. As indicated by Liu et al. [4], the remelting depth can be of high importance for the prevention of cracks and should be larger than the heat-affected zone of the subsequent DMD layer. Therefore, the remelting depth is of higher relevance for process optimization, and is further investigated within this study.

**Figure 9:**
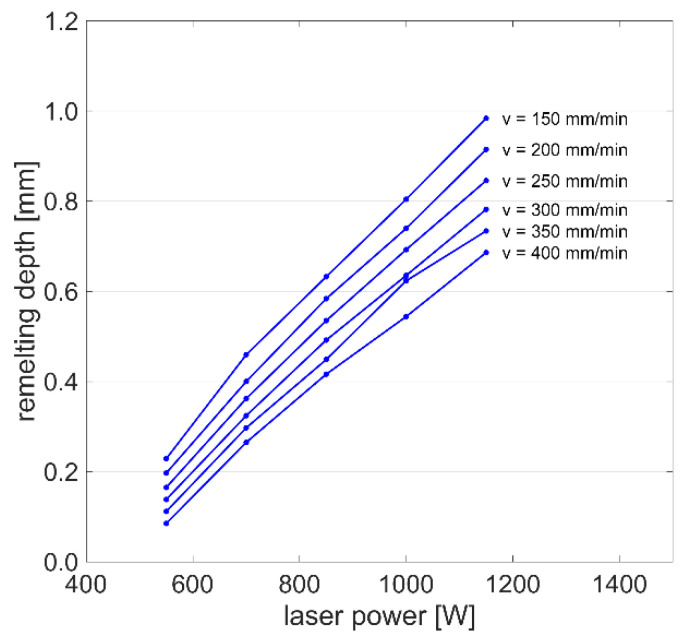
Remelting depth as a function of laser power and scanning speed with constant subtrate temperature of 0 °C, obtained by the heat conduction model.

Figure 9 presents the remelting depth as a function of laser power and scanning speed as calculated by the heat conduction model. The depth of the melt pool rises nearly linearly with increased laser power *P* and reduced scanning speed *v*, which is similar to the findings of Afrasiabi et al. [20]. The results confirm that once the heat source provides sufficient energy to melt the material, an increase of the line energy

(11) $$e_{L}=\frac{P}{v}$$

generally leads to larger melt pool dimensions.

**Figure 10:**
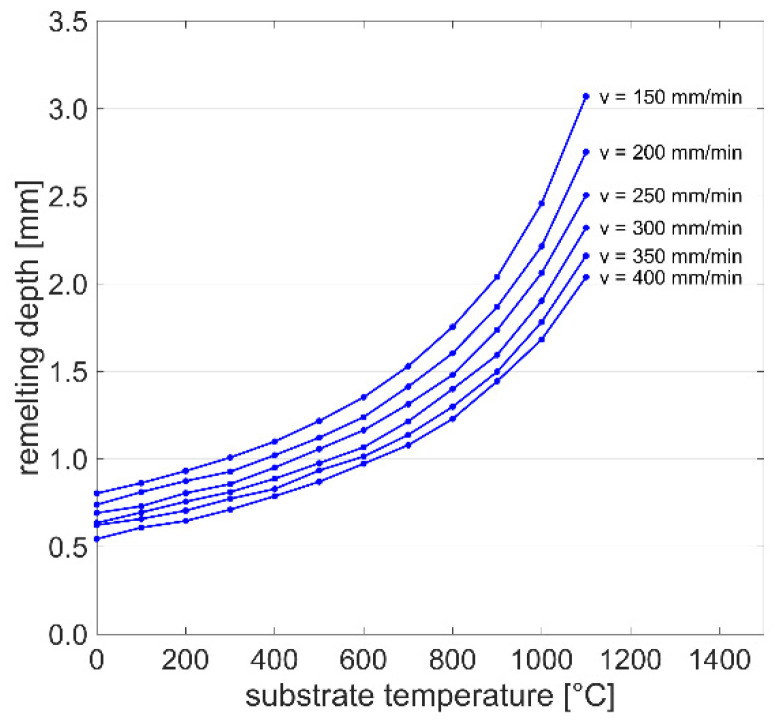
Remelting depth as a function of substrate temperature and scanning speed with constant laser power of 1000 W, obtained by the heat conduction model.

Figure 10 shows the effect of the initial substrate temperature on the remelting depth for six scanning speed levels and a constant laser power of 1000 W. The melt pool depth *d* monotonically increases with the substrate temperature *T*, and the correlation with the scanning speed *v* can be described by

(12) $$d=A_{1}\left(e^{A_{2}T}+A_{3}\right)\left(v+A_{4}\right)$$

with *A*_1_ = −1.832 × 10^−4^, *A*_2_ = 2.476 × 10^−3^, *A*_3_ = 4.72, and *A*_4_ = −948.70, which leads to a determination coefficient *R*^2^ of 0.996. These results are of high interest for applications with multiple welding tracks, especially in the field of metal additive manufacturing, where substrate overheating occurs frequently [21]. The quantitative description of this effect can help to develop compensation strategies to keep the melt pool dimensions more stable, which increases the robustness of the deposition process.

**Figure 11:**
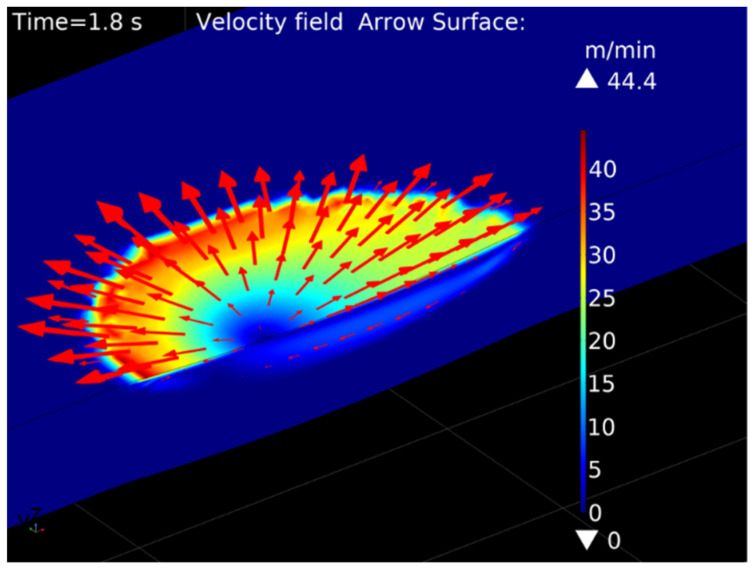
Velocity field of melt pool motion, computed by the fluid flow model for *P* = 1000 W, *v* = 400 mm/min, *T* = 20 °C.

The velocity field within the melt pool computed by the fluid flow model is visualized in Figure 11. The velocity magnitude can be up to two orders of magnitude larger than the scanning speed. The liquid metal flows from the central to the peripheral area on the top surface, then downwards back to the central area. As shown by Knapp et al. [8], surface temperature gradients lead to gradients in surface tension and thereby to Marangoni stresses, which are the main drivers of convective flow within the melt pool. The calculated velocity fields in the present study confirm that the Marangoni effect is the dominant driving mechanism in melt pool motion.

### 4.2. Single Track Validation

**Figure 12:**
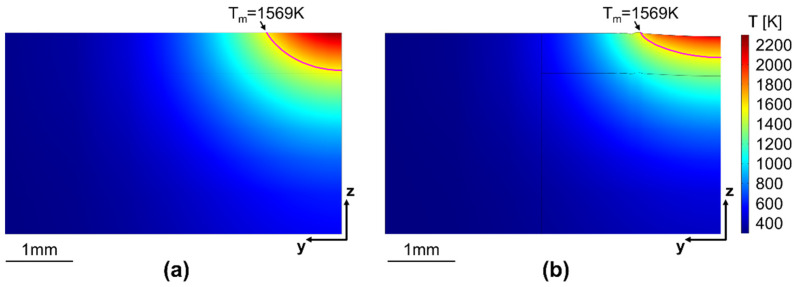
Temperature fields within the model domain and isothermal curves at remelting temperature *T_m_* calculated by (**a**) the heat conduction and (**b**) the fluid flow model for *P* = 1000 W, *v* = 400 mm/min, *T* = 20 °C.

Figure 12a,b show the temperature fields for one reference condition calculated by the heat conduction and fluid model, respectively. The melt pool geometries are approximated by the isothermal curves at the remelting temperature *T_m_*, which is assumed to be the arithmetic mean value between the solidus and liquidus temperatures of the material. The geometries predicted by the two models are significantly different from each other, as the consideration of fluid flow results in a wider and shallower melt pool.

**Figure 13:**
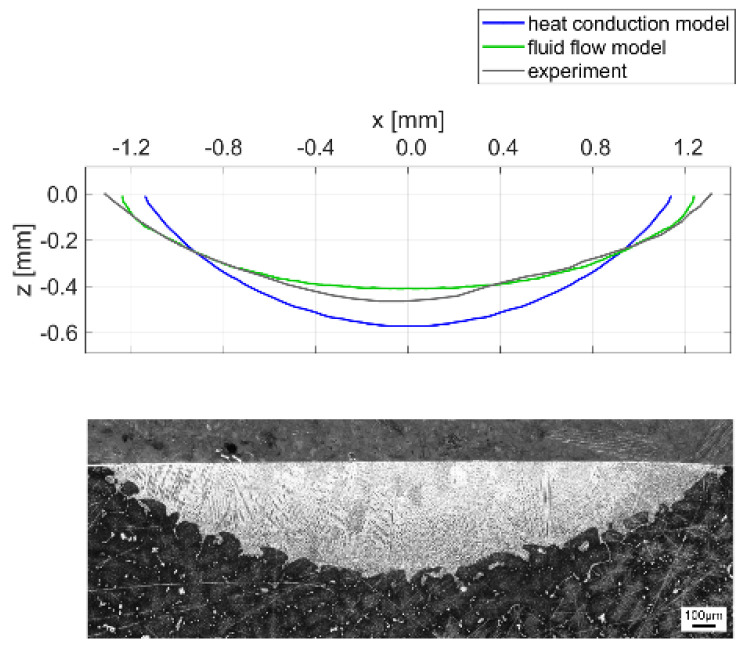
Comparison of the melt pool geometries obtained by two different numerical approaches and the experimental result for *P* = 1000 W, *v* = 400 mm/min, *T* = 20 °C.

Figure 13 compares the melt pool geometries predicted by the two numerical models with the experimental results. While the heat conduction model slightly overestimates the remelting depth and underestimates the width, the fluid flow model predicts the actual geometry very well for this reference condition. According to Le and Lo [22], the outward flow pattern in Figure 11 can be related to a negative surface tension gradient and leads to a wider and shallower melt pool. Hence, the fluid motion within the melt pool significantly affects the final shape of the remelted zone, and the surface tension model provided by Mills and Su [17] and described in Equation (9) increases the accuracy of the numerical model. While the predicted melt pool geometries are very smooth, the interface in the experimentally-obtained cross section shows a patterned structure. This is due to the dendritic microstructure of the cast base material and the segregation of phases with a lower melting point in the interdendritic regions. As the melt pool dimensions were determined by the best fit along the interface, the assumption of a remelting temperature between solidus and liquidus temperatures appears suitable for this study.

**Figure 14:**
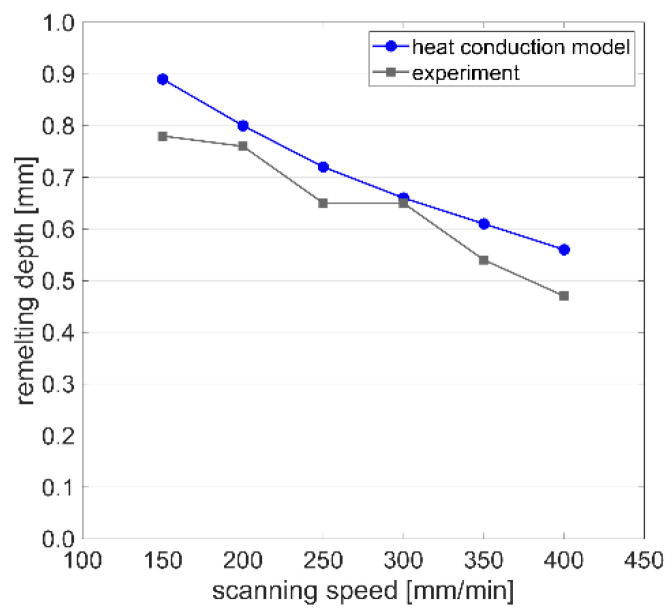
Comparison of the remelting depth computed by the heat conduction model with the experimental results for *P* = 1000 W and *T* = 20 °C with scanning speeds varying from 150 to 400 mm/min.

Further experimentally-obtained melt pool cross sections are compared to the simulation results in Figure 14. The heat conduction model predicts the remelting depth with deviations between +1.5 and +19.1%. Thus, the results computed by the heat conduction model can be considered as sufficiently accurate for the purposes of process understanding and optimization.

**Figure 15:**
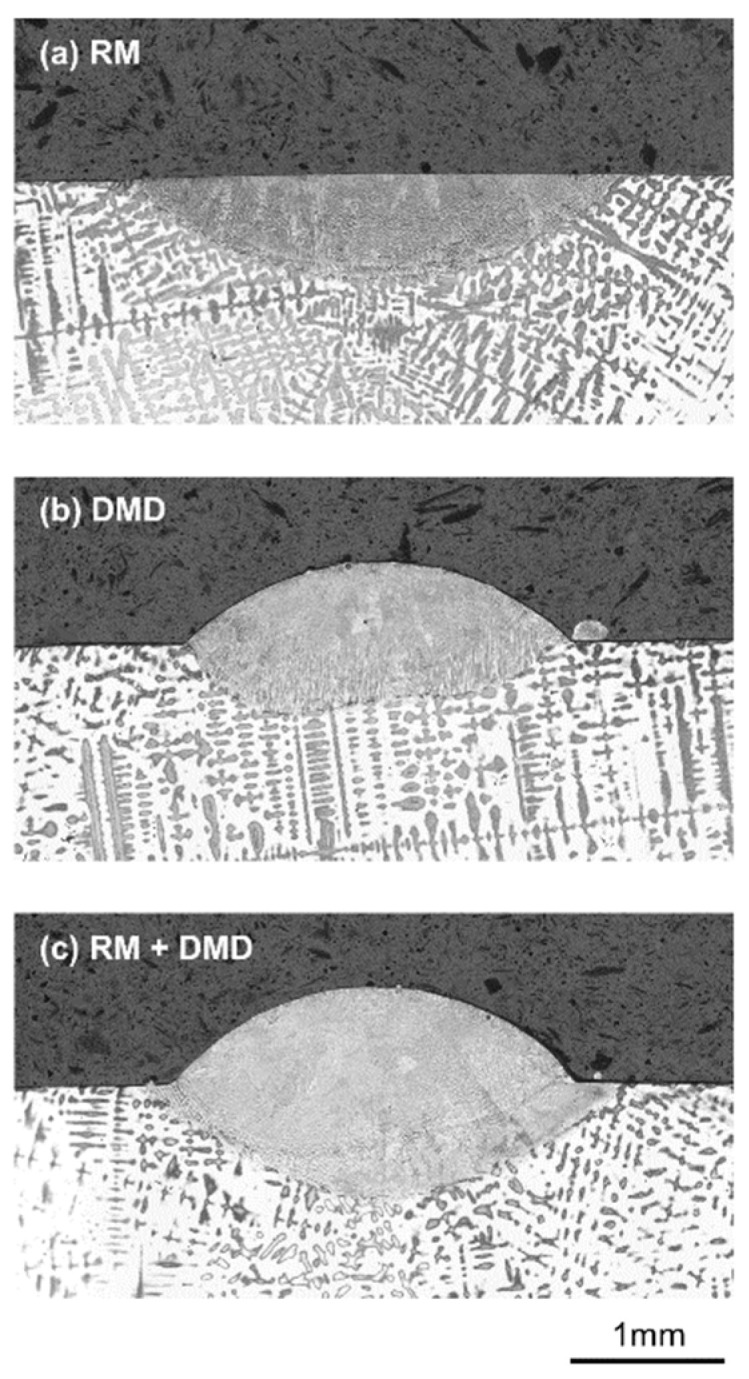
Single track geometries for (**a**) laser remelting only (*P* = 1000 W, *v* = 250 mm/min), (**b**) DMD only (*P* = 1000 W, *m* = 4.0 g/min), (**c**) laser remelting and DMD.

Figure 15 shows the effect of a laser remelting track on the substrate bonding of a subsequently-deposited DMD track. While the remelting track in Figure 15a has a regular and smooth bonding line, the DMD track without prior remelting in Figure 15b shows irregular bonding to the substrate. This irregular bonding might be explained by unsymmetrical powder feeding, as described by Eisenbarth et al. [23]. For the DMD track with prior remelting in Figure 15c, the regular and smooth substrate bonding remains unchanged, and it is apparent that the maximum remelting depth is larger than the dilution caused by the DMD process. Hence, the selected remelting parameters fulfill the required purpose for these DMD process conditions.

### 4.3. Groove Repair Process Optimization

**Figure 16:**
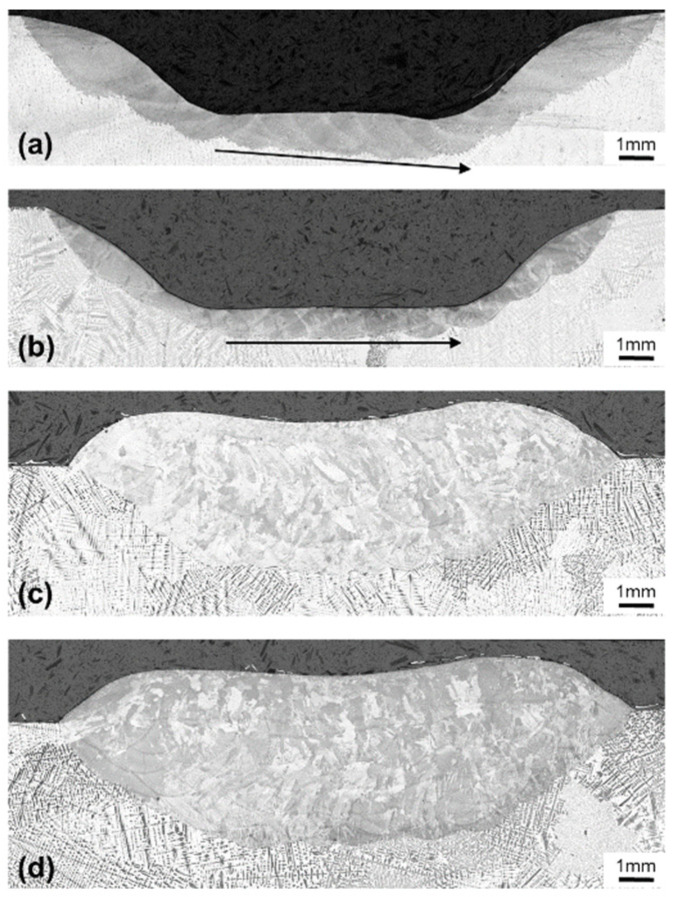
Groove cross sections after (**a**) laser remelting with constant scanning speed (*v* = 300 mm/min), (**b**) laser remelting with variable scanning speed (*v* = 250–400 mm/min), (**c**) DMD groove filling only without prior remelting, and (**d**) laser remelting with variable scanning speed and DMD groove filling.

The laser remelting process was applied to the groove geometry as described in Section 3. Remelting with a constant scanning speed of 300 mm/min was evaluated with the metallographic cross section shown in Figure 16a. In the bottom section, where the laser remelts the material with eight raster paths, the remelting depth increases linearly. This is due to heat accumulation, which leads to an increased substrate temperature. The low heat conductivity of Inconel 718 increases the risk of substrate overheating, especially for parts with small cross-sections and multiple short welding tracks adjacent to each other. The remelting depth of the individual raster tracks in the groove bottom is between 1.03 mm and 1.32 mm. Thus, substrate overheating leads to an increase in the remelting depth of 28%. Compared to the heat conduction model results in Figure 10 for the scanning speed of 300 mm/min, such an increase of remelting depth could be related to a 300 K substrate temperature increase. This finding is similar to the observations by Higashi and Yoshimi [24], where multi-track scanning increased the substrate temperature, and therefore the size of the resulting melt pools. To realize a more constant remelting depth, the remelting process can be optimized via several methods. One option is to include cooling times after each deposited track, which would lead to an increased processing time. Another option is to reduce the amount of heat input, either by reducing the laser power or increasing the scanning speed. In order to optimize the laser remelting process for both a stable remelting depth and minimum processing time, the option with increased scanning speed was selected. Figure 16b shows the result of laser remelting with a scanning speed that was increased from 250 mm/min for the first raster path to 400 mm/min for the last, along with the resulting contour paths. Overall, the remelting depth is much more stable compared to the specimen in Figure 16a. Figure 16c,d shows the cross sections of grooves filled by DMD without and with prior laser remelting. Similar to the findings of Zhang et al. [25], there is a high bonding quality for both conditions, without bonding defects. The specimen with prior laser remelting shows a more regular bonding depth, especially in the inclined wall area. Thus, the optimized laser remelting process may be applied for repair applications that require very regular substrate bonding. Furthermore, the combined milling and laser processing machine allowed for the realization of all process steps within one setup, which is of advantage for the dimensional accuracy of the repair weldment.

For the qualification of AM processes, it is essential to analyze the mechanical properties of the deposited material. A previous study of the authors [26] investigated the microstructural evolution and tensile properties of Inconel 718 deposited by DMD without laser remelting on cast IN718 substrates. Here, tensile testing of the interface specimens showed that the interface is not the critical zone under tensile load, as the fracture location was within the cast section, where the material has a lower yield strength due to its larger grains. Therefore, cast Inconel 718 seems not to require laser remelting prior to the DMD process for the investigated conditions. The main limitations of the present study are related to the numerical accuracy of the fluid flow model. While the heat conduction model could be solved very quickly for a large amount of processing conditions, it slightly overestimated the remelting depth and underestimates the width. In contrast, the fluid flow model could predict the melt pool shape of one reference condition very accurately; however, the high computing time and limited model robustness prevented its application for detailed parameter studies.

Overall, both of the numerical models developed were able to predict the melt pool geometries of laser remelting tracks. Single track and groove filling experiments with and without remelting confirm that the laser remelting process increases the bonding quality. This improved bonding quality may lead to greater durability of parts fabricated or repaired using DMD.

## 5. Conclusions

The present study describes two numerical models for the simulation of laser remelting. Single track experiments were performed for model validation along with part scale experiments, demonstrating the applicability of the developed remelting process for metal additive manufacturing. The results confirm that the proposed process increases the quality of metal AM parts. The simulation and experimental results lead to the following conclusions:

- The neglection of fluid flow in the numerical simulation reduces the computing time from 16 h to less than one minute.
- The simplified heat conduction model is useful for quantifying the effects of the main laser remelting processing parameters.
- Single remelting track experiments with varying scanning speeds confirm the high physical accuracy of both the heat conduction model and the fluid flow model. The fluid flow model showed the highest geometrical accuracy, while the heat conduction model slightly overestimated the remelting depth and underestimated the width.
- Single tracks fabricated by DMD with and without prior remelting show that the remelting step leads to more uniform substrate bonding.
- The application of laser remelting within a process chain for part repair confirms increased bonding quality. Furthermore, the remelting process is expected to be suitable for defect prevention in metal AM part fabrication and repair.

## Figures and Tables

**Table 1:** Constant material properties of Inconel 718 for the numerical simulations according to Pottlacher et al. [12] and Anderson et al. [16].

Property	Symbol	Value	Source
Density	*ρ*	8190 kg/m^3^	[12]
Solidus temperature	*T_s_*	1528 K	[12]
Liquidus temperature	*T_l_*	1610 K	[12]
Melting enthalpy	*h_m_*	227,000 J/kg	[12]
Coefficient of thermal expansion	*β*	6.473 × 10^−5^ K^−1^	[12]
Work piece absorptivity	*α_wp_*	0.3	[16]

**Table 2:** Process conditions for the full-factorial parameter study.

Parameter	Symbol	Unit	Values
Laser power	*P*	W	550, 700, 850, 1000, 1150
Scanning speed	*v*	mm/min	150, 200, 250, 300, 350, 400
Substrate temperature	*T*	°C	0–1100 in steps of 100

## Data Availability

The datasets generated during and/or analyzed during the current study are available from the corresponding author on reasonable request.
